# Comment on Wang et al. Consumption of JUUL vs. Other E-Cigarette Brands among U.S. E-Cigarette Users: Evidence from Wave 5 of the PATH Study. *Int. J. Environ. Res. Public Health* 2022, *19*, 10837

**DOI:** 10.3390/ijerph20186715

**Published:** 2023-09-06

**Authors:** Floe Foxon, Saul Shiffman

**Affiliations:** PinneyAssociates Inc., Pittsburgh, PA 15213, USA; shiffman@pinneyassociates.com

This comment is to express concern about an article by Wang et al. [1], in which the authors purport to show that, compared to use of other e-cigarette brands, use of the JUUL brand of e-cigarette by adolescents was associated with lower odds of current smoking and greater odds of dependence (defined as wanting to first use e-cigarettes within 30 min of waking). That analysis depended on data from Wave 5 of the Population Assessment of Tobacco and Health (PATH) survey.

We attempted to replicate the findings using the same PATH survey data and the analytic methods prescribed by PATH and found that the analysis produced different results. Specifically, the above differences between use of JUUL and other brands reported in the Wang et al. paper were not statistically significant, contrary to the published report. We contacted the authors, and they provided us with their statistical analysis code. A review of that code showed that the analysis was not done in a manner consistent with the guidance from the PATH survey team. In particular, they did not take the prescribed steps to account for PATH’s complex sampling design. The result of Wang et al.’s reliance on incorrect analytic approaches is that their conclusions with regard to adolescent JUUL users are incorrect. We shared these concerns with the authors of the Wang et al. paper, who suggested that we submit them to the journal.

In brief, the PATH survey uses a complex, four-stage, stratified probability sample design, and this was not taken into account in the Wang et al. analysis. PATH explicitly provides guidance on the methods and explicit Stata code that “should be used” (as dictated in the PATH User Guide [2], pp. 47, 142, 144). That guidance was not followed in the Wang et al. analysis. The code used to analyze the PATH data departed from the prescribed approach in two material ways: (1) It did not take replicate weights into account, and (2) it did not use the version of logistic regression that accommodates complex survey design (i.e., the missing replicate weights). Technical details on the PATH-recommended analysis and the analysis that underpinned the published paper by Wang et al. are made available in supplemental material (https://doi.org/10.17605/OSF.IO/QVPWH (accessed on 17 May 2023)).

Wang et al., in their response, emphasize that they took into account *sampling* weights to adjust the sample to be nationally representative. We agree that that was handled appropriately. They also claim that this took account of the entire complex sampling design. That is crucially incorrect because the authors did not include *replicate* weights in their analyses. The complex sampling design involves multi-stage sampling, where individual respondents are sampled from larger units. In contrast to the *sampling* weights, where misspecification impedes the representativeness of the results arising from unequal probabilities of selection, the *replicate* weights affect the variance estimation of the data, with the result that the variance is not correctly estimated when the *replicate* weights are not taken into account. According to Lewis [3], pp. 226, “replicate weights… provide all of the complex design information necessary to properly calculate a variance”. A mis-estimation of the variance can, in turn, affect conclusions about statistical significance. The authors point to other papers that may also have failed to account for the sampling design by not using replicate weights. That others may have made this same error does not remedy the problem. Whether that affected the conclusions in those other analyses, we do not know. But in the case of the analysis by Wang et al., it did affect the conclusions. In the article by McMillen et al. [4] cited by Wang et al., the stratum and cluster variables in the PATH dataset were used instead of the replicate weights. The Wang et al. analysis used neither the replicate weights *nor* the stratum and cluster variables, which is why their estimation of variance is problematic.

Wang et al., in their response, also suggest that the version of logistic regression they used is mathematically equivalent to the version recommended by the PATH survey team. However, that is only true when replicate weights are excluded from the analysis. The two procedures are *not* equivalent when replicate weights are used, as they should have been, following PATH guidance.

The impact of these departures from the PATH User Guide is material. Whereas in the original analysis by Wang et al., the odds ratios of current smoking and dependence in adolescents using JUUL compared to other e-cigarette brands were statistically significant, when the analysis is done appropriately following PATH’s guidance (using the code provided by the PATH survey team) and guidance in statistics texts (e.g., [5,6]), these odds ratios are not statistically significant (i.e., confidence intervals crossing 1; notably, applying the correct method to the young adult and adult analyses also changed the results in detail, but not in a way that changes the conclusions).

Thus, the departure from the analytic approach prescribed by PATH led the authors to incorrectly conclude that JUUL use was associated with lower odds of smoking and higher odds of dependence among adolescents. Those conclusions are not supported when the analyses are correctly executed.

We appreciate the authors’ openness in sharing their code and the journal for giving us the opportunity to comment.

## Data Availability

The data used in this research are available on the Population Assessment of Tobacco and Health website (https://www.icpsr.umich.edu/web/NAHDAP/studies/36231 (accessed on 10 March 2023)).
